# Identifying the opportunities and barriers: a cross-sectional review of the health development coordination mechanisms in the World Health Organization African Region

**DOI:** 10.3389/fpubh.2025.1559742

**Published:** 2025-08-06

**Authors:** Abdulmumini Usman, Olushayo Oluseun Olu, Mohamed Kakay, Ndoungou Salla Ba, Patrick Kabore, Hyelni Kulausa, Emmanuel Chanda, Alex Gasasira, Joseph Cabore

**Affiliations:** ^1^World Health Organization African Regional Office, Brazzaville, Republic of Congo; ^2^World Health Organization Country Office, Kabul, Afghanistan

**Keywords:** health sector coordination, World Health Organization, WHO African Region, policy brief, Africa

## Abstract

Effective health sector coordination is essential to improve the implementation of development and humanitarian response programmes in the WHO African Region. In recent years, multiple efforts have been deployed to strengthen coordination mechanisms, with uneven progress depending on the national context. Nevertheless, important challenges remain. In many cases, mechanisms remain fragmented, coordination platforms often operate in isolation, and resources are not always used efficiently. In certain settings, current cooperation models may hinder the long-term sustainability of progress, and there is a need to foster greater synergy among the actors involved. Strengthening national capacities and promoting coordination that better responds to realities on the ground therefore remain critical priorities on the continent. While several studies have assessed coordination mechanisms in individual countries, a regional overview has been lacking. This study thus aimed to examine the current status and characteristics of health sector coordination mechanisms and the role of WHO in health coordination across the 47 member states of the WHO African Region. A descriptive cross-sectional study was conducted between 2017 and 2019 using a mixed-methods approach, including document reviews, key informant interviews, and focus group discussions. The findings revealed that only 9 member states (23%) had a functioning central health sector coordination platform, all of which had signed the IHP + compact and adopted the Sector-Wide Approach. Nearly half (47%) lacked a central platform, despite 41% hosting a significant number of development partners and 36% having signed the IHP+. Sixteen member states (34%) operated multiple fragmented, programme-specific coordination mechanisms with minimal linkage to any overarching platform, despite most of them (81%) being IHP + signatories. In eight member states, parallel humanitarian and development coordination systems coexisted. Coordination was identified by stakeholders as one of the top five priorities for WHO support. Based on these findings, five key recommendations are proposed: strengthening national coordination capacities; streamlining multiple coordination mechanisms; addressing coordination challenges in protracted crisis settings; clearly defining WHO’s coordination role in line with partner expectations; and providing context-specific support across core health system functions. Collective efforts from all stakeholders are essential to accelerate the implementation of these recommendations, thereby enhancing health governance, improving aid effectiveness, and advancing progress towarduniversal health coverage in the region.

## Introduction

Coordination is the unification, integration, synchronization of the efforts of group members to provide unity of action in the pursuit of common goals, as an integral element or ingredient of all the managerial functions (1). In the context of this paper, its goal is to improve aid effectiveness and reduce transaction costs in view of the multiplicity of donors, projects, and programmes covering multiple countries (2). While ensuring that programme beneficiaries are reached with high-quality services (3).

Partner coordination remains a complex task, often shaped by differing priorities between donors and recipient countries. While donors tend to operate in line with their institutional mandates and strategic objectives, recipient countries seek to ensure that external support aligns with national plans and upholds their sovereign decision-making over public policy (4). It is therefore essential to align the interests of all stakeholders around common goals, and to foster environments that promote sustained coordination while preventing what is often referred to as “coordination fatigue.” (5). Existing coordination mechanisms within the health sectors such as the health sector coordinating groups are attempts to provide overall sector coordination, while others aim to address specific interest or achieve project/programme specific objectives. The Paris Declaration on Aid Effectiveness and the subsequent Accra Agenda for Action (6) were adopted to improve country ownership, harmonization, alignment, and mutual accountability of development aid which ultimately should result in more effective aid coordination. Additionally, the International Health Partnership and Related initiatives (IHP+) was established in 2007 to strengthen health development aid globally (7).

The coordination needs of many countries include alignment of development aid and interventions with national priorities, coordination of funding streams, and management of data to support evidence-based programme planning, implementation and evaluation (8). In the health sector, the emergence of major donors and the Global Health Initiatives (GHIs) such as the Global Fund for AIDS, Tuberculosis and Malaria (GFATM) and the Global Vaccine Alliance (Gavi) and their model of channeling significant amounts of highly earmarked resources through Non-governmental Organisations (NGOs) and United Nations (UN) agencies have improved access to health care services. Nevertheless, this aid delivery model has, in some contexts, contributed to and added layer of coordination resulting in persistent coordination challenges and an uneven use of available resources. This highlights the urgency of moving toward more integrated and sustainable coordination mechanisms in the health sector (9, 10).

The challenges arising from the presence of multiple donors and GHIs with multiple aid delivery and coordination mechanisms include the existence of parallel aid planning and coordination processes, the inequitable distribution of health workers, in some cases influenced by salary imbalances across programmes (11), lack of sustainability and increased dependency on foreign aid (12). These dynamics may erode governance structures at national and subnational levels, hinder transparency and accountability processes, and ultimately impact governmental leadership, oversight, and coordination capacity in the health sector (13). Other aid coordination challenges include duplication of supply chain procurements and management systems (14), malalignment of intergovernmental and health sector-specific coordination mechanisms and verticalization of health programmes particularly disease prevention programmes (15). These challenges often lead to difficulties in sustaining and expanding coverage of priority health services by national health systems (7).

Health coordination is among the key functions the World Health Organisation (WHO) is expected to perform especially global stewardship in setting norms, provision of policy and operational support to countries, and global health governance using its convening power (16). The organization’s 13th General Programme of Work (GPW 13), a 5-year strategy which ran from 2019 to 2024, highlighted support to coordination of health sector partners among its priorities (17). Given that many MSs of the WHO African Region (WHO/AFR) largely rely on international cooperation support (18), health coordination is often complex and requires several elements that need to work together to improve aid effectiveness (17). Consequently, there have been calls for better coordination of donors and GHI support by WHO in ways that comprehensively cover more priorities and contribute toward strengthening the health systems at the country level (19). For instance, in Mozambique, some bilateral donors have promoted sector coordination (20). Also, Mongolia has made progress attributable to a holistic approach to strengthening governance through building capacities including for planning and donor coordination (21).

However, experiences at strengthening health coordination at country level have demonstrated mixed results. Attempts to harmonize and coordinate support resulted in only 14% of the overall health funding being channeled through the coordination system (22). In Kenya, duplications and fragmentations continued despite the existence of a formal coordination structure (15). A case study covering Bangladesh, Cambodia, Mozambique, Zambia, and South Africa, showed that for health coordination to be impactful, it should go beyond the coordination mechanism to strengthening the whole system (23). The effectiveness of the coordination will also be suboptimal if relevant sectors are not involved as shown by the impact of climate change on health in Vietnam (24).

While some studies have examined health sector coordination in specific African countries, none has reviewed the situation across the region (25, 26). Furthermore, very few have reviewed both health development and humanitarian coordination mechanisms. This study therefore aims to review the status and nature of current health sector coordination mechanisms in WHO/AFR and to describe the perceived roles of WHO in the health sector coordination and strategies to improve donor coordination in WHO/AFR countries. The evidence provided by this study may be useful for regional public health organizations, donors, and national health authorities seeking to strengthen their health development coordination mechanisms. Moreover, as these processes consolidate, the resulting models could become transferable, further reinforcing the strategic value of the African Region’s field-based experience.

## Methods

### Study design

This is a descriptive cross-sectional study using a mixed-method design involving document review, key informant interviews (KIIs) and focus group discussions (FGDs) in the 47 MSs of the WHO/AFR between 2017 to 2019.

### Study setting

WHO/AFR comprises 47 African MSs^1^ that are largely located in sub-Saharan Africa. Despite progress in areas such as immunization and infectious diseases control, the region continues to experience significant public health challenges. As of 2022, the neonatal mortality rate in the Region was 26 per 1,000 live births, and the under-five mortality rate was 70 per 1,000 live births, figures which are significantly higher than those of the other WHO regions (27). The same applies to the maternal mortality ratio which stood at 531 deaths per 100,000 live births in 2020 (27). Similarly, the region has some of the largest burden of infectious diseases globally. For example, in 2022, the incidence of tuberculosis per 100,000 population was 208 in 2022 (second only to the Southeast Asia region) while the malaria incidence stood at 223 per 100,000 population which is the highest globally (27). Due to changing lifestyle, nutrition, environmental and other factors, the region has continued to experience an increasing incidence of non-communicable diseases resulting in a double burden of diseases.

Unfortunately, the health system capacity in the region remains low due to poor planning, weak health governance and oversight, underfunding and the effect of chronic conflicts and humanitarian crises which affects several African countries. The density of health workers, which is a key determinant of health services delivery, is low. The physicians and midwifery personnel per 10,000 population were 2.6 and 11.3 which are far below the recommended ratio.

In 2018, the average government health expenditure as a percentage of total government expenditure in the African Region ranged from 2.2% in the Republic of Congo to 10% in Central Africa Republic which are below the 15% target set by the Abuja Declaration (27). With such low domestic health financing levels, foreign development aid in the form of direct budget support and specialized bilateral and multilateral funding mechanisms remain the main sources of healthcare financing in the region. Additionally, out-of-pocket expenditure as a percentage of total health expenditure in the region ranged from 4.4% in Botswana to 76% in Nigeria, indicating a high financial burden on individuals and households (27). With a Universal Health Coverage (UHC) service coverage index of 44 out of 100 as of 2021, the region lags behind the global average of 68 and those of other WHO regional blocks such as the Eastern Mediterranean region that is 57 and South-East Asia that is 62 (27).

### Study participants

The WHO Regional Office for Africa (WHO/AFRO), the secretariat of WHO/AFR is headed by a Regional Director and supported by WHO Representatives, who are responsible for overseeing the organisation’s operations across the 47 MSs in the region. At the country level, WHO/AFRO offices implement the organisation’s core functions, which include providing technical assistance to MSs in the adaptation and implementation of international and regional health norms and standards, as well as supporting governments in convening and coordinating health sector partners. These functions are undertaken in collaboration with a range of bilateral and multilateral stakeholders, including national ministries of health, UN agencies, international and national non-governmental organisations, civil society actors, and donor agencies. The study participants were drawn from this broad spectrum of health development stakeholders. A purposive sampling technique was used to collect information from a diverse group of stakeholders. Hence, a formal sample size calculation for a normal quantitative study was not strictly required for data collection. Data was collected until saturation was reached. The participants included Ministers of Health or their designate, typically Permanent Secretaries or senior technical directors, as well as heads of health development cooperation within donor agencies, country directors of international and national NGOs, and heads of relevant UN agencies. These individuals are typically key actors in health development and humanitarian coordination processes, working in close partnership with WHO to support effective health governance, coordination and service delivery in their respective countries.

### Data collection

#### Document review

A range of country-level documents were collected and analysed using a standardized template to obtain a comprehensive overview of the health coordination landscape across MSs. These documents included national health situation analyses, national health policies, health sector strategic plans, WHO Country Cooperation Strategies and their respective mid-term reviews, as well as other relevant national and partner-generated materials. The standardized template was designed to systematically capture key elements including contextual background, health system status, strategic directions and priorities, the partnership landscape, particularly health sector coordination arrangements, as well as an assessment of WHO country office capacities, encompassing both human and financial resources and operational modalities.

#### Key informant interviews and focus group discussions

KIIs were conducted with Ministers of Health or their designated representatives, who were predominantly Permanent Secretaries or the most senior officials responsible for international health coordination within the Ministries of Health of all 47 WHO African Region MSs. In addition, FGDs were held with other key stakeholders, organized into two distinct groups: members of the United Nations Country Teams and representatives of health development partners. Semi-structured interview and discussion guides were developed to facilitate both KIIs and FGDs all of which were conducted in person. These guides included questions designed to elicit participants’ understanding of the roles and functions of WHO, perceptions of what the organisation can and should do, and the priority deliverables and outputs expected by governments and their key partners. The guides also aimed to identify the roles played by other health partners in order to inform WHO’s potential engagement and alignment with existing coordination structures and collaborative frameworks. Detailed notes were taken during all interviews and discussions by the study team and subsequently transcribed for analysis.

#### Data analysis

A member of the survey team extracted information from the documents using a template to identify and categorize countries according to the existence and type of coordination mechanisms. The detailed descriptions and terms of reference of the various types of coordination mechanisms were also summarized. The information was then organized into thematic areas showing the countries’ situations, including in the area of coordination. This was shared with other team members of the research team for review and validation. The notes from the KIIs and FGDs were grouped into themes and concepts concerning the role of WHO in coordination. Summary counts of each theme/concept were generated, and percentages were calculated and presented in a tabular form.

#### Ethical clearance

Ethical clearance for the study was requested from the WHO/AFRO Ethics Review Committee. The Committee granted an exemption from formal ethical review, as the study was part of an internal organizational reform process and did not present any anticipated risk to human participants. The participation of key informants was voluntary, and confidentiality was ensured in line with the ethical principles applicable to research of this nature.

## Results

### Document review

The results of the document review are presented in Tables 1, 2. They show that at the time of the study, only 9 (19.1%) WHO/AFR MSs had a fairly functioning overall central sector coordination platform with 8 (88.9%) of them having signed the IHP + and are implementing the Sector-Wide Approach (SWAP). About half (47%) did not have a central coordination platform, despite 9 (41%) of them having significant partner presence with 8 (36%) having signed the IHP+. Sixteen MSs (34%) had multiple verticalized, programme/project-based coordination mechanisms with little or no link to the existing central coordination platform, despite the majority (81%) having signed the IHP+. There were vertical humanitarian and development coordination systems in 8 (17%) MSs (Table 1).

**Table 1:** Health sector coordination arrangements at country level.

	No	Percent age	Names of Countries
No formal central coordination platforms (some vertical coordination mechanisms may exist)			
Total Countries (*n* = 47)	22	46.8%	Algeria, Angola, Botswana, Cape Verde, Congo, Equatorial Guinea, Eritrea, Eswatini, Gabon, Gambia, Guinea Bissau, Madagascar, Mauritius, Mozambique, Namibia, STP, Senegal, Seychelles, South Africa, South Sudan, Togo, Zimbabwe
Significant Partner Presence (*n* = 22)	9	40.9%	Angola, Cape Verde, Equatorial Guinea, Eswatini, Guinea Bissau, Madagascar, Mozambique, Togo, Zimbabwe
Signed IHP + (*n* = 22)	8	36.4%	Congo, Gambia, Guinea Bissau, Mozambique, Senegal, South Africa, Togo, Zimbabwe
Fairly functional coordination mechanism			
Total Countries (*n* = 47)	9	19.1%	Burkina Faso, Cote d’Ivoire, Ghana, Kenya, Liberia, Malawi, Rwanda, Uganda, Zambia
Signed IHP + (*n* = 9)	8	88.9%	Burkina Faso, Cote d’Ivoire, Ghana, Kenya, Liberia, Rwanda, Uganda, Zambia
Multiple coordination with no/poor link to central coordination mechanism or vertical humanitarian/development			
Total Countries (*n* = 47)	16	34.0%	Benin, Burundi, Cameroun, CAR, Chad, Comoros, DRC, Ethiopia, Guinea, Lesotho, Mali, Mauritania, Niger, Nigeria, Sierra Leone, Tanzania
Signed IHP + (*n* = 16)	13	81.3%	Benin, Burundi, Cameroon, Chad, Comoros, DRC, Ethiopia, Guinea, Mali, Mauritania, Niger, Nigeria, Sierra Leone
Vertical Coordination Mechanisms for Humanitarian and Development Support			
Total Countries (*n* = 47)	8	17.0%	CAR, DRC, Ethiopia, Mali, Mozambique, Niger, Nigeria, South Sudan

**Table 2:** Existing mechanisms for health sector coordination at country level.

Mechanism	Description	Examples	Functions
High level health governance coordination forums	Health Sector Dialogue Framework/Joint Sector Review forum brings together all health sector stakeholders to engage in policy dialogue; and to ensure ownership, accountability and transparency of the national strategy/HSSP IV implementation and monitoring process including through Annual Sector Planning and Review	Compact between the Government and its development partners	Compact aims to improve the economic and social effectiveness of aid and promote sustainable health development by strengthening the leadership of MOH in defining, managing, monitoring and evaluating national health policy based on the Aids Effectiveness
		Health Sector Coordinating Groups (HSCG) and other high level coordination mechanisms generally chaired by the Ministers of health. Such as and Joint Interagency Coordination Committee (JICC), Health, HSSP National Steering Committee, Inter-Agency Leadership Committee (IALC)	Provides a forum to achieve effective engagement with all health sector stakeholders to conduct a coordinated coherent and quality policy dialogue among health sector stakeholders; and provide input to the formulation of sector policies, strategies and programs and to ensure coherence with the national development plan among other functions
Partners’ coordination mechanisms	These groups chaired or co-chaired by one of the partners is open to bilateral and multilateral partners operating in health and usually excludes the government. However, government officials are usually invited to participate in discussions around a particular topic when necessary.	Health Partners’ Consultation Forum, Health Development Partner’s Forum, Health Donor Group, PTF Santé in Francophone countries	To foster harmonization of partner support to government priorities, foster common understanding of health sector issues and communicate as one to the government, promote reduction of transaction costs for government, contribute to the development of sector policies, strategies and plans and endeavors to consolidate inputs and comments as a group
UN Coordination Mechanisms	An attempt by the UN agencies to work around common development agenda	UN Country Teams (UNCT)	To coordinate The UN agencies’ inputs to health through UNDAF/UNSDF and monitored by the UNCT and its various organs
		H4/H6 group of partners (WHO, UNICEF, UNFPA, UNAIDS, World Bank and UN Women)	To focus and advocate for reproductive, maternal and child health.
Humanitarian Coordination	Brings together all stakeholders including UN agencies, local and international NGOs and donors that provide humanitarian health response	Humanitarian Health Cluster	Is responsible for the development of the Health Humanitarian Response Plan as well as mobilization of resources, coordination, monitoring and evaluation of its implementation
Global Health Initiatives Coordination Mechanisms		The Country Coordination Committee of the Global Fund to Fight AIDS, Tuberculosis and Malaria (CCM)	Assures the coordination and the follow-up of the execution of the programs approved by the Global Fund and evaluates the performance of the principal recipients of the grants.
		EPI Inter Agency Coordination Committee (ICC)	Validates and monitors the EPI annual action plans, coordinates interventions of the various partners in immunization, mobilizes resources for immunization, and evaluates the implementation of the EPI plan
Technical working groups	Technical working groups (TWGs) are operational entities where technical and policy issues are discussed by staff of the MOH with relevant and interested representatives of development partners, NGOs, and FBOs.	Sector Working Group (SWG), Inter-agency Coordinating Committees (ICCs), Disease specific working groups	Provide the platform for discussing technical issues on specific themes/diseases that will feed into main coordination mechanisms
Sub-national level coordination	Serve as coordination mechanisms at the regional and district levels	Subnational Health Cluster, Regional Dialogue Frameworks, District Health Stakeholder Committee	To support coordination of activities at the sub national level for both development and humanitarian interventions

The document review further showed the existence of multiple coordination arrangements for the health sector WHO/AFR MSs. These included (i) High level health governance coordination forums that bring together all health sector stakeholders; (ii) Partners’ coordination mechanisms open to bilateral and multilateral partners operating in health and usually excludes the government; (iii) UN coordination mechanisms, an attempt by the UN agencies to work around common development agenda; (iv) Humanitarian coordination mechanisms that bring together all stakeholders that provide humanitarian health response; (v) GHI coordination mechanisms; (vi) Technical working groups that discuss technical and policy issues; and (vii) Sub-national level coordination mechanisms which serves as coordination mechanisms at the regional and district levels. The descriptions and functions of these mechanisms are presented in Table 2.

### Key informant interviews and focus group discussions

More than 300 KIIs and FGDs were held in 47 countries. The most mentioned expected coordination function required of WHO includes to ensure the presence and active participation of partners in a functional coordination mechanism (89%), generation of evidence to guide interventions (89%), normative guidance and coordination of technical assistance (83%) and promotion of intersectoral action (77%) (Table 3). Other highly required coordination functions included strengthening capacities for governance (70%), strengthening subnational level coordination (70%), monitoring of accountability (53%) advocacy (51%) and alignment of health interventions to national priorities (40%) (Table 3).

**Table 3:** Coordination functions expected from WHO in countries.

S/N	Situation	No	% (*n* = 47)
1	Establishment/strengthening of/active participation in Existing Coordination Mechanisms	42	89%
2	Generation and dissemination of evidence to guide planning and implementation of health interventions	42	89%
3	Improving normative guidance and coordination of technical assistance	39	83%
4	Promoting intersectoral action	36	77%
5	Strengthening capacities for health governance	33	70%
6	Strengthening subnational health coordination	33	70%
7	Stronger monitoring of mutual accountability	25	53%
8	Advocacy	24	51%
9	Alignment of health interventions to national priorities	19	40%
10	Facilitation of south–south cooperation partnerships	8	17%

Three main thematic areas which align with the foregoing quantitative results emerged from the qualitative analysis of the KIIs and FGDs. First, there was a consensus that health coordination was ineffective in most African countries as a result of several parallel platforms, there were differentiated expectations of WHO’s coordination role. While both governments and partners acknowledge the importance of WHO in health sector coordination, their expectations diverge slightly. Governments expect WHO to play a stronger role in holding partners accountable In contrast, partners view WHO as a neutral broker that facilitates engagement and collaboration with government counterparts. Second, government and partners expected WHO to build institutional capacity at both national and subnational levels, including partner mapping and coordination structures, to reduce duplication and enhance value for money in health investments. Third, the was a strong demand for WHO to support the establishment and operationalization of national health coordination platforms. These platforms, they said should be geared toward facilitating policy dialogue, monitor the impact of strategies, and ensure more efficient, accountable coordination of technical and financial support across the health sector.

## Discussion

This study aimed to assess the current status and characteristics of health sector coordination mechanisms in the WHO/AFR, and to analyse the perceived role of WHO in strengthening such coordination. The findings indicate that many WHO/AFR MSs still lack well established formal mechanisms for central health sector coordination, despite the presence of multiple development partners and global and regional frameworks designed to support such coordination. In countries where specific coordination platforms do exist, these often present limitations in terms of operational capacity, coherence, and alignment among actors, which hampers fully effective collaboration. Additionally, the study revealed that in countries affected by protracted conflict, the coexistence of humanitarian and development coordination structures posed a considerable challenge to effective health system recovery and resilience building. The partners interviewed called for improved engagement, capacity and involvement of WHO in strengthening health coordination functions of its African MSs using a differentiated approach (26).

The generally weak and ineffective health development coordination which was observed in this study may be attributed to a number of factors such as inadequate health coordination capacity within the government structure, the presence of multiple partners and coordination mechanisms, lack of adherence to the aspirations of the Paris declaration on harmonization and alignment and the Accra agenda and the presence of humanitarian-development divide (HDD). This is buttressed by the findings a of a recent study in Ghana and Sierra Leone that showed that strengthening coordination requires the mobilization of country level actors and skilled leadership (28). Similarly, it was observed that the vertical mechanisms of some GHIs are not always fully integrated into national coordination systems, which may result in overlaps, misaligned priorities, and fragmentation across the health system (20). This disconnect could hamper coherent progress toward UHC and the achievement of the Sustainable Development Goals (SDGs). This is further buttressed by other studies such as a WHO study in the Democratic Republic of the Congo (DRC) (29).

The study findings further show that even in countries where coordination mechanisms exist, they did not function optimally calling for the reorganization and strengthening of such mechanisms. In countries with fairly functional coordination mechanisms such as Cote d’Ivoire and Burkina Faso, more emphasis on the evidence generation, articulation of evidence-based policy options and monitoring and evaluation is required to further strengthen these mechanisms. This also applies to Uganda and Rwanda, where governments have demonstrated commitment to the principles of the Paris Declaration on Aid Effectiveness and the Accra Agenda for Action through the signing of IHP + compacts with development partners. These agreements are intended to enhance the effectiveness and alignment of external assistance in the health sector. In other contexts, such as Algeria, high-level strategic guidance could help enhance the impact of the development partners currently active in the country. A similar need may be observed in countries like Cabo Verde, Mauritius, and São Tomé and Príncipe, where many health development activities are coordinated from neighboring countries. In such settings, strengthening the technical presence and mechanisms for sustained engagement may contribute to a more integrated and context-responsive coordination process.

HDD was identified as critical health coordination challenges in MSs affected by protracted conflicts. The existence of parallel health development and humanitarian coordination mechanisms in such settings hinders post-conflict health systems recovery and resilience building (30). While the humanitarian health clusters seek to improve the effectiveness of humanitarian response by ensuring greater predictability, accountability and partnership (31), they are often vertical and separated from the development coordination mechanisms due to their nature (32). Nevertheless, the value of the humanitarian health clusters in improving health outcomes and reducing crude morbidity and mortality in humanitarian settings is critical (32). Thus, bridging the HDD in such settings to advance health system recovery and sustainable development agenda is an imperative. This should be done by ensuring close collaboration between health development and humanitarian coordination platforms and ensuring the transition of the humanitarian coordination mechanisms into development coordination at the end of the humanitarian emergencies (32). In this regard, chronic-affected MSs such as the Central African Republic and DRC will require innovative approaches and consistent interventions to bridge the HDD. Additionally, the active participation of the health sector coordination mechanisms in the health cluster activities and ensuring synergy between humanitarian programming and coordination is important in such settings (32).

Recognizing the health coordination gaps and challenges and the aspirations of WHO/AFR MSs and health partners to improve health coordination in the region, WHO country offices in the region need to strengthen their capacity for health coordination and play a more active role in health coordination. In this regard, a few key roles are proposed (Table 4). These proposals are based on a few considerations. First, more emphasis should be placed on strengthening the coordination of health planning and policy development to enhance national ownership and partners alignment as observed by Walt and Gibson (33). This is also in line with the assertions of Walt et al. (23) that addressing comprehensive sector strategies has a better impact on the system than discrete project-based coordination supported management units. Second, although health coordination contexts differ from one country to another, the overall objective is to strengthen health systems for UHC (34). It is therefore important to optimize existing coordination mechanisms toward achieving this objective, as demanded by stakeholders in almost 90% of the countries during the reviews. Third, achieving UHC requires health financing reforms aimed at improving health objectives at the population or system levels (35). Thus, health financing should be a critical pillar in health coordination to facilitate sustainable and equitable generation, allocation and efficient use of financial resources. Fourth, strategic health information is necessary for evidence-based health decision-making, policy appraisal, planning, implementation and evaluation and efficient use of resources. It should therefore be included in health coordination. Additionally, capacity strengthening in data analytics to guide resource allocation and contribute to improved efficiency in the use of resources is also critical. Finally, given that most of the existing multiple and vertical coordination mechanisms are linked to service delivery, a coordination function that integrates them to ensure efficiency and effectiveness is important.

**Table 4:** Proposed coordination functions for WHO country offices.

Coordination area	Objective	Proposed key WHO role
Policy/Planning	To provide a tool for national ownership and partner alignment	Provide evidence to shape National Agenda Facilitate policy dialogue toward sound policies Coordinate technical support to develop a solid national costed multisectoral health plan
Coordination mechanism	To provide a forum for harmonization of inputs and stakeholder dialogue	Facilitate the establishment/effective functionality of a national health dialogue forum Broker on issues between national authorities and other stakeholders based on evidence Maintain and updated comprehensive partner map to promote inclusiveness Facilitate dialogue between humanitarian and development actors
Monitoring and evaluation	To generate evidence for mutual accountability	Facilitate development of sound monitoring and evaluation Frame for National Health Plan Facilitate generation of evidence Facilitate Annual Sector Review between Government and Partners
Financing	To ensure equity in resources allocation and promote efficiency	Support the preparation of National Health Accounts Maintain an updated resource mapping and gap analysis to facilitate equitable resources shifting
Service delivery	To promote efficiency and integration	Facilitate the design and implementation of essential integrated health packages Promote the bridging of the humanitarian/development nexus

The findings of this study should be interpreted in the context of a few limitations which are associated with descriptive and region-wide studies. The sampling for selection of the respondents was purposive, which could have introduced some biases that a random sampling would have eliminated. However, the sampling method is acceptable for KIIs and FGDs. The analysis is primarily based on key informant interviews and group discussions, which may reflect perceptions shaped by context or individual experience. The study does not explore qualitative dimensions such as political dynamics or organizational culture, which are also relevant to coordination mechanisms. Most authors are affiliated with UN system institutions, which may have influenced the framing of some aspects. Additionally, as data were collected between 2017 and 2019, some coordination structures may have evolved since then, particularly in the context of the COVID-19 pandemic which emerged in 2020. Future studies could benefit from complementary approaches that include comparative methodologies and statistical tools to support deeper analysis and inform evidence based policy decisions.

## Conclusion

This study assessed the status and challenges of health sector coordination across WHO/AFR MSs and examined the role of WHO in strengthening coordination efforts. The findings suggest that, in several MSs, several opportunities remain to strengthen national platforms for health sector coordination. For instance, despite the presence of multiple development partners and regional and global frameworks that should provide an enabling environment, many countries still lack centralized and fully functional coordination structures. Where such platforms exist, they are often configured in a vertical and limited manner, underscoring the need for more integrated coordination approaches. The coexistence of parallel humanitarian and development coordination structures posed notable challenges for sustainable recovery of health systems in countries affected by protracted crises. These structural and functional challenges are hindrances to the efficient delivery of cost-effective, high-quality health services and will continue to impede progress toward the achievement of UHC in the region.

Based on the study’s findings, we propose five main recommendations. First, the coordination capacities of WHO/AFR MSs should be systematically strengthened. Second, advocacy should be intensified to streamline the multiplicity of coordination platforms and mechanisms operating across the region. Third, HDD and the coordination challenges in countries affected by protracted conflicts should be addressed and bridged. Fourth, WHO’s role in health sector coordination should be more clearly defined and aligned with partner expectations and country-specific contexts. This role should extend beyond the establishment of coordination structures to encompass support for coordination of critical health system functions, including policy and strategy development, health financing, service delivery, and strategic information management. Furthermore, WHO’s coordination support should be context-sensitive, recognizing and adapting to the diversity of coordination needs, mechanisms, and tools across different settings in the region. Finally, the active participation of all public health stakeholders in the Region is would be essential to advance the implementation of the recommendations outlined in this study, reinforce collective health coordination and governance efforts, and contribute to improved health outcomes, which is undoubtedly the shared objective of all involved.

## Data Availability

The original contributions presented in the study are included in the article/supplementary material, further inquiries can be directed to the corresponding author.

## Footnotes

^1^Algeria, Angola, Benin, Botswana, Burkina Faso, Burundi, Cameroon, Cape Verde, Central African Republic, Chad, Comoros, Ivory Coast, Democratic Republic of the Congo, Equatorial Guinea, Eritrea, Ethiopia, Gabon, Gambia, Ghana, Guinea, Guinea-Bissau, Kenya, Lesotho, Liberia, Madagascar, Malawi, Mali, Mauritania, Mauritius, Mozambique, Namibia, Niger, Nigeria, Republic of the Congo, Rwanda, São Tomé and Príncipe, Senegal, Seychelles, Sierra Leone, South Africa, South Sudan, Eswatini, Togo, Uganda, Tanzania, Zambia, Zimbabwe.
